# Crosslinked Polymer Ionic Liquid/Ionic Liquid Blends Prepared by Photopolymerization as Solid-State Electrolytes in Supercapacitors

**DOI:** 10.3390/nano8040225

**Published:** 2018-04-07

**Authors:** Po-Hsin Wang, Tzong-Liu Wang, Wen-Churng Lin, Hung-Yin Lin, Mei-Hwa Lee, Chien-Hsin Yang

**Affiliations:** 1 Department of Chemical and Materials Engineering, National University of Kaohsiung, Kaohsiung 81148, Taiwan; p5802341@gmail.com (P.-H.W.); tlwang@nuk.edu.tw (T.-L.W.); linhy@nuk.edu.tw (H.-Y.L.); 2 Department of Environmental Engineering, Kun Shan University, Tainan 71070, Taiwan; linwc@mail.ksu.edu.tw; 3 Department of Materials Science and Engineering, I-Shou University, Kaohsiung 84001, Taiwan; mhlee@isu.edu.tw

**Keywords:** supercapacitor, polymer ionic liquid, ionic liquid, solid electrolyte, photopolymerization

## Abstract

A photopolymerization method is used to prepare a mixture of polymer ionic liquid (PIL) and ionic liquid (IL). This mixture is used as a solid-state electrolyte in carbon nanoparticle (CNP)-based symmetric supercapacitors. The solid electrolyte is a binary mixture of a PIL and its corresponding IL. The PIL matrix is a cross-linked polyelectrolyte with an imidazole salt cation coupled with two anions of Br^−^ in PIL-M-(Br) and TFSI^−^ in PIL-M-(TFSI), respectively. The corresponding ionic liquids have imidazolium salt cation coupled with two anions of Br^−^ and TFSI^−^, respectively. This study investigates the electrochemical characteristics of PILs and their corresponding IL mixtures used as a solid electrolyte in supercapacitors. Results show that a specific capacitance, maximum power density and energy density of 87 and 58 F·g^−1^, 40 and 48 kW·kg^−1^, and 107 and 59.9 Wh·kg^−1^ were achieved in supercapacitors based on (PIL-M-(Br)) and (PIL-M-(TFSI)) solid electrolytes, respectively.

## 1. Introduction

Green power generation and energy storage have been the most attractive subject of research in recent years. Supercapacitors are an energy storage device. Supercapacitors are often used in high power output or energy storage systems such as starters, hybrid cars and uninterruptible power supplies [1,2,3].

Supercapacitors have electric double layer capacitors (EDLC) and pseudocapacitors. The former uses a physical method (Coulombs static force) to store charges, whereas the latter uses a chemical method (redox reaction) to store the charges. Asymmetric devices are generally composed of the pseudocapacitor anode [4] and the electric double layer cathode. The main materials are carbon materials, metal oxides, and conductive polymers. Carbon is the most common electrode material for supercapacitors due to low cost, high specific surface area, good conductivity, and high chemical stability. Carbon materials are mainly used in EDLC, but functional groups are involved on their surface that would lead to the appearance of pseudo-capacitance characteristics [5]. 

In supercapacitors, the electrolyte needs to provide a sufficient concentration of positive and negative ions, good ionic conductivity, and no chemical reaction with the packaging material. In addition, the electrolyte also determines the operation potential window of a supercapacitor. In carbon-based supercapacitors using 1 M sulfuric acid and 6 M potassium hydroxide [6] at a charge–discharge current density of 0.04 A·g^−1^, the potential windows only reached 0.6 and 1 V, and the specific capacitances of 242 and 208 F·g^−1^, respectively. The potential operated above 1.23 V will cause water to be decomposed, insinuating the potential-window limitation of 1 V in the aqueous electrolyte. Moreover, aqueous electrolytes often contain a strong acid (H_2_SO_4_) and base (KOH), leading to electrode corrosion. Organic electrolyte has a higher operating potential (about 2 V) in relation to the aqueous system, so that this system can store more charge. In addition, organic electrolyte not only has lower corrosion than the aqueous electrolyte, but also less reactivity with the electrode. However, organic electrolyte has a relatively high resistance resulting from poor ion conductivity. Ionic liquids (ILs) have a wide range of operating potential window that allow them to store more charges with a higher energy density, chemical stability at high temperature, and environmental friendliness. Solid-state electrolytes are mostly prepared by incorporating polymers in a liquid electrolyte. The ionic conductivity of the solid electrolyte is lower than that of the above two systems, mainly because the ion transport in the solid electrolyte is hard, causing a large internal resistance, and the separator can be omitted in the device.

Ionic liquid can be prepared with the desired characteristics according to different needs, because it has a great variety of cation and anion combinations [7]. Izmaylova et al. [8] used 1-methyl-3-butylimidazolium tetrafluoroborate ([MBIM] [BF_4_]) as the electrolyte in a carbon-based symmetric supercapacitor, obtaining a specific energy density and specific power density of 4.1 Wh·kg^−1^ and 1.7 Wk·g^−1^, respectively. A symmetric supercapacitor was made using activated carbon fiber cloth as the electrodes and mixed 1-ethyl-3-methylimidazolium bromide and 1-ethyl-3-methylimidazolium tetrafluoroborate as the electrolyte ([EMIm] [Br]/[EMIm] [BF_4_]). The potential window was up to 2 V, and the specific capacitances were 59 F·g^−1^ at a charge–discharge current density of 0.1 A·g^−1^. The performance of electrolyte-containing bromide ion is better than the electrolyte without bromide ion [9]. However, there is a drawback—electrolyte leakage—which makes IL extremely troublesome to package the supercapacitors. To solve this problem, a solid or quansi-solid state electrolyte is substituted for IL. In 2014, a polymer electrolyte of tetramethoxysilane and formic acid was prepared employing the sol–gel reaction and then mixed with 1-ethyl-3-methylimidazolium bis(trifluoromethanesulfonimide) ([EMIm] [TFSI]), which has been utilized in a carbon-based symmetric supercapacitor with a potential window of 3 V at a charge–discharge density of 1 A·g^−1^ [10] and the specific capacitance of 48 F·g^−1^ with 100% capacitance retention after 10,000 cycles. Poly (ionic liquids) (PILs) are made by the polymerization of corresponding ionic liquid monomers. Most PILs are a polycation in which the cationic groups bear on the polymer main chain. Except for common pyridinium, pyrrolidonium, and imidazolium, there still are vinyl, styrenic, and methacrylic groups bearing on the monomers through free radical polymerization to obtain the corresponding polymers. Recently, cross-linked poly-4-vinylphenol (c-P4VPh) was employed as a polymer matrix and mixed 1-ethyl-3-methylimidazolium bis(trifluoromethylsulfonyl) imide ([EMI] [TFSI]) as a polymer electrolyte, which was involved in a porous carbon-based symmetric supercapacitor [11] with a potential window of 4 V at a charge–discharge density of 1 mA·cm^−2^ and a specific capacitance of 172.45 F·g^−1^, an energy density of 72.26 Wh·kg^−1^, and a power density of 1696.56 W·kg^−1^. More recently, photopolymerization was employed to polymerize vinylimidazole monomers containing bromine anions in PILs [12,13,14], obtaining non-crystalline polymers which are very suitable for use as solid electrolytes [14]. 

In this work, we hope to prepare a solid electrolyte instead of the liquid electrolyte and separator in a carbon nanoparticle (CNP)-based supercapacitor. We chose carbon nanoparticle (CNP) as the electrode material of the supercapacitor due to low cost and stability. To enhance the ionic conductivity of solid electrolytes, a binary component of IL soaked into PIL was employed as a solid state electrolyte. Here, butane-substituted vinylimidazolium salt, 1-methyl-3-butylimidazolium as the cation and bromide as the anion, was photopolymerized to form a PIL. This has the advantages of good thermal stability, high electrochemical stability and high ionic conductivity to achieve a high performance carbon nanoparticle (CNP)-based supercapacitor. To increase the potential window enhancing the energy density, the bromide anion was ion-exchanged by bis(trifluoromethane sulfonamide anion (TFSI^−1^) in ionic liquid, whereas a cross-linked PIL was prepared as the solid electrolyte with high mechanical strength, retaining more IL.

## 2. Materials and Methods

### 2.1. Chemicals

1-Bromobutane (Alfa Aesar, Tewksbury, MA, USA), 1,4-dibromobutane (Alfa Aesar, Tewksbury, MA, USA), 1-vinylimidazole (Alfa Aesar, Tewksbury, MA, USA), 1-methylimidazole (Alfa Aesar, Tewksbury, MA, USA), lithium bis(trifluoromethane sulfonamide) (LiTFSI, Acros, Geel, Belgium), photoinitiators: diphenyl (2,4,6-trimethylbenzoyl)-phosphine oxide (TPO, Aldrich, Milwaukee, WI, USA), 1-hydroxy-cyclohexyl-phenyl-ketone (Irgacure 184, Ciba, Tarrytown, NY, USA), 2-methyl-1-[4-(methylthio)phenyl]-2-(4-morpholinyl)-1-propanone (Irgacure 907, Ciba, Tarrytown, NY, USA), poly(vinylidene fluoride) (PVDF, Solf^®^PVDF 6020, Solvay, Bruxelles, Belgium), 1-methyl-2-pyrrolidone (NMP, Riedel-de Haën, Seelze, Germany), carbon nanoparticles (CNP, ~15 nm, UniRegion Bio-Tech, Taoyuan, Taiwan), and activated carbon (AC, ACS-2930, China steel chem. Co., Kaohsiung, Taiwan) were used as received.

### 2.2. Preparation of Ionic Liquid

The chemical reactions involved in the preparation of the materials are sketched in Table 1.

#### 2.2.1. Preparation of Ionic Liquid Monomer

The general synthetic procedures for ionic liquid monomers (ILMs) followed the literature [15]. To prepare 1-methyl-3-butylimidazolium bromide (MBIB), 0.1 mole of 1-methylimidazole, 0.1 mole of 1-bromobutane and 30 mL of methanol were loaded into a 100 mL reactor. The mixture was stirred at 60 °C for 15 h. After cooling down, the reaction mixture was added dropwise into 1 L of diethyl ether to extract the product. A yellow liquid product (1-methyl-3-butylimidazolium bromide, MBIB) was collected and completely dried at 60 °C under vacuum. 

To prepare 3-*n*-butyl-1-vinylimidazolium bromide (BVIB), 0.1 mole of 1-vinylimidazole was used to replace 1-methylimidazole. The other chemicals were the same as for the preparation of MBIB, and the preparation procedure also followed the preparation of MBIB. 

To prepare 1,4-butanediyl-3,3’-bis-l-vinylimidazolium dibromide (BDVIB), 0.2 mole of 1-vinylimidazole and 0.1 mole of 1,4-dibromobutane were used to replace 0.1 mole of 1-methylimidazole and 0.1 mole of 1-bromobutane, respectively. Other chemicals were the same as the preparation of MBIB, and the preparation procedure also followed the preparation of MBIB.

To prepare MBIT, 0.01 mole of MBIB, 0.01 mole of LiTFSI, and 8 mL of distilled water were mixed and stirred in a three-necked flask at 80 °C for 24 h under nitrogen purge. Then, 15 mL of distilled water solution was added to the above reaction solution and kept stirring to cool at room temperature. The solution was extracted using CH_2_Cl_2_ to extract, and then repeatedly washed with distilled water. A yellow liquid product was obtained after being set in a vacuum oven for 24 h. 

The preparation procedures of BVIT were the same as that of MBIT rather than MBIB by VBIB. The preparation procedures of BDVIT were the same as that of MBIT rather than MBIB by BDVIB and using 0.02 mole of LiTFSI. 

#### 2.2.2. Preparation of PIL

To prepare crosslinked PIL-(Br), 16.77 mmole of BVIB, 2.93 mmole of BDVIB, 150 mg of mixed photoinitiators (60 mg TPO + 45 mg Irgacure 184 + 45 mg Irgacure 907), and 1.5 g of acetone were mixed at 50 °C until completely dissolved. Acetone in the mixture was removed by purging with dry argon at 60 °C for 24 h. After cooling down to room temperature, the reaction mixture was cast on a 50 cm^2^ area polyimide plate. This thin IL film was exposed to ultraviolet light irradiation of 125 W (emission peak at 365 nm, Ausbond) keeping a distance of 10 cm from the film to the mercury bulb for 10 min, the power level was 8 mW/cm^2^ measured at 365 nm, performing photopolymerization to generate a film product on the polyimide substrate. The hypothesized molecular structures of PIL-(Br) and PIL-(TFSI) are proposed in Table 1.

To prepare the PIL/IL blend (PIL-M-(Br)), 4.3 mmole of MBIB, 16.77 mmole of BVIB, 2.93 mmole of BDVIB, 150 mg of mixed photoinitiators (60 mg TPO + 45 mg Irgacure 184 + 45 mg Irgacure 907), and 1.5 g of acetone were mixed at 50 °C until completely dissolved. Acetone in the mixture was removed by purging with dry argon at 60 °C for 24 h. After cooling down to room temperature, the reaction mixture was cast on a 50 cm^2^ area polyimide plate. This thin IL film was exposed to ultraviolet light irradiation of 125 W (emission peak at 365 nm, Ausbond) keeping a distance of 10 cm from the film to the mercury bulb for 10 min, the power level was 8 mW/cm^2^ measured at 365 nm, performing photopolymerization to generate a film product on the polyimide substrate.

The preparation procedures of crosslinked PIL-(TFSI) were the same as that of crosslinked PIL-(Br) rather than BDVIB by BDVIT and VBIB by VBIT, respectively. The preparation procedures of PIL/IL blend (PIL-M-(TFSI)) were the same as that of PIL/IL blend (PIL-M-(Br)) rather than BDVIB by BDVIT, VBIB by VBIT, and MBIB by MBIT, respectively. 

### 2.3. Electrode Preparation

Graphite paper (20 × 10 mm) was ground to a smooth surface using coarse and fine sandpaper in a sequence, washed using 0.5 M sulfuric acid to clean the surface, and then transferred to dry in the oven at 120 °C for 2 h obtaining a graphite-paper current collector. Activated carbon, carbon nanoparticles, and PVDF were mixed and ground in an agate bowl with a weight ratio of 7:1:1, and then an appropriate amount of NMP was added and pulverized to a slurry paste. An appropriate amount of the paste was dropped on the polish graphite paper (10 × 10 mm) and spin-coated to form a uniform film. Then the above film was baked in a circulation oven at 70 °C for 24 h to obtain the carbon electrode. The symmetric capacitors were sandwiched by two working electrodes that were separated by polymer ionic liquid/ionic liquid (PIL-M-(Br) or PIL-M-(TFSI)) blend films as the electrolyte and separator with a thickness of ca. 0.25 mm. IL electrolytes of MBIB and MBIT were injected into a bipolar CNP-electrode cell with a porous polypropylene separator (~0.25 mm thickness).

### 2.4. Characterization and Measurements

Infrared spectra were recorded on a Fourier transform infrared spectroscopy (FTIR) spectrometer (Agilent Technologies, Cary 630, Santa Clara, CA, USA) to check the functional groups on polymer films using an attenuated total reflection (ATR) cell. Film surface morphology was observed on field emission scanning electron microscopy (FESEM, Hitachi, S4800, Tokyo, Japan). The PIL film samples were put on aluminum film prior to imaging. ^1^H nuclear magnetic resonance (^1^H NMR) measurements were carried out at room temperature using a Bruker AV400 FT-NMR spectrometer (Billerica, MA, USA) operating at 400 MHz. Chloroform-d_1_ or dimethylsulfoxide (DMSO)-d_6_ were used as solvents. Differential scanning calorimetry (DSC) measurements were performed on a TA instrument (SDT-Q600, New Castle, DE, USA). The samples were first heated up to 100 °C, and then the samples were cooled from +100 to −50 °C at a cooling rate of 10 °C/min in a cooling process, where they were kept at −50 °C for 2 min. Finally, the samples were reheated to 295 °C at a heating rate of 10 °C/min. The glass transition point of PILs was determined by the heating curve. Thermal gravitational analysis (TGA) was performed on a TA instrument (SDT-Q100, New Castle, DE, USA). 

### 2.5. Electrochemical Characterization

All of the electrochemical experiments were investigated using an AUTOLAB PGSTAT302N electrochemical work station (Metrohm Autolab, Utrecht, The Netherlands). A typical three-electrode cell, filled with electrolyte and equipped with a working electrode, platinum foil as a counter electrode and an Ag/AgCl reference electrode, was employed to measure the electrochemical properties of the AC-CNP carbon-coated working electrode and its symmetric device at 25 °C. The cyclic voltammograms (CVs) of the AC-CNP carbon-coated electrode were recorded by varying the scan rates: 5, 10 mV·s^−1^ over the potential range of −0.2–0.8 V for the three-electrode cell and 10, 25, 50, 75, 100 and 125 mV·s^−1^ over the potential range of 0–0.8 V for the symmetric supercapacitors. Measurements of electrochemical impedance spectroscopy (EIS) were carried out on the bipolar CNP-electrode cell using an alternating current (AC) bias of 0.1 V with a signal of 10 mV over the frequency range of 0.1 Hz to 100 kHz at 25 °C. Solid-electrolyte films (~0.25 mm thickness) of PIL-M-(Br) and PIL-M-(TFSI) were directly sandwiched between the bipolar CNP-electrodes. IL electrolytes of MBIB and MBIT were injected into a bipolar CNP-electrode cell with a porous polypropylene separator (~0.25 mm thickness). The galvanostatic charge–discharge curves were obtained over the potential range of 0–3.0 V for various current densities (0.5, 1, 1.5, 2, 2.5, 5 and 10 A·g^−1^) in symmetric devices. A current density of 1, 5 A·g^−1^ was used in the symmetric cell.

## 3. Results and Discussion

### 3.1. Morphology of PILs Derived from Photopolymerization

**Figure 1:**
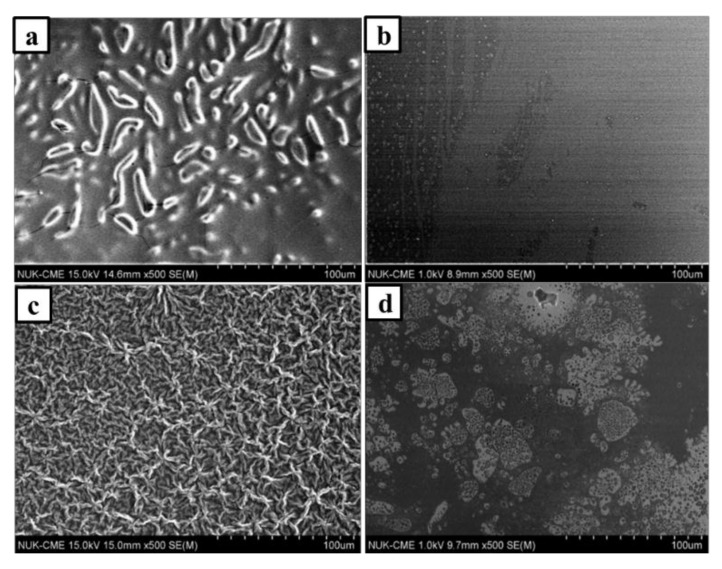
Scanning electron microscopy (SEM) images of (**a**) polymer ionic liquid (PIL)-(Br); (**b**) PIL-M-(Br); (**c**) PIL-(TFSI); and (**d**) PIL-M- (TFSI) films.

Figure 1a shows SEM image of the PIL-(Br) film, exhibiting an uneven surface with fine cracks and bumps. These features could be due to shrinking upon polymerization and thus suggest the presence of considerable attractive intermolecular interactions between polymeric chains in the forming films. The SEM image of the PIL-M-(Br) film exhibited a smoother surface with a few fine holes in comparison with the compared PIL-(Br) film as shown in Figure 1b. This result suggests that the fine drops of MBIB liquid phase are present in the generating polymer film during photopolymerization in the binary mixture of BVIB (Br^−^), BDVIB (Br^−^), and MBIB (Br^−^). On the other hand, a cross-linked network structure of PIL-(TFSI) polymer can be observed with a uniform distribution of tangled lines in Figure 1c. Figure 1d shows the image of PIL-M-(TFSI), exhibiting a smooth surface with many holes of different sizes. This result implies that the MBIB liquid drop phase is also present in the generating polymer film during photopolymerization in the binary mixture of BDVIB (TFSI^−^), BDVIB (TFSI^−^), and MBIB (TFSI^−^). 

### 3.2. FTIR Spectra of PILs Derived from Photopolymerization

**Figure 2:**
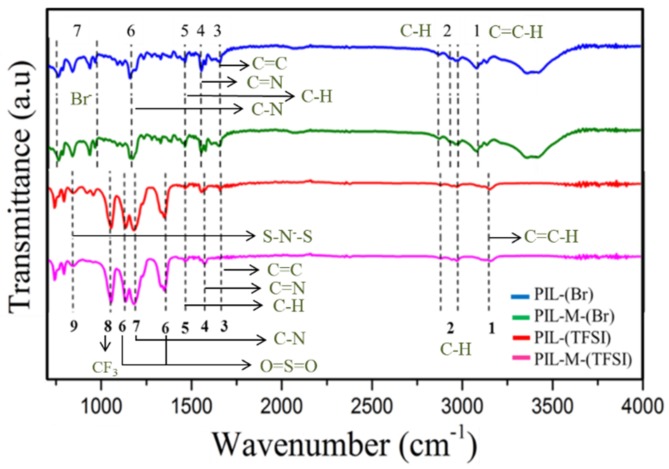
Fourier transform infrared spectroscopy (FTIR) spectra of PIL-(Br), PIL-M-(Br), PIL-(TFSI), and PIL-M-(TFSI).

Figure 2 shows the FTIR spectra of PIL-(Br), PIL-M-(Br), PIL-(TFSI), and PIL-M-(TFSI) and Table 2 lists the assignment of functional groups above the polyelectrolytes. The stretching of C–H (SP^2^) presents at 3077 cm^−1^, which is contributed by the C=C–H bond on the imidazole ring. The stretching of C–H (SP^3^) presents at the three peaks of 2981, 2932 and 2869 cm^−1^, corresponding to the C–C–H bond on the imidazole ring. The stretching of C=C and C=N on the aromatic ring presents at 1650 and 1546 cm^−1^, corresponding to the C=C and C=N bonds, respectively, on the imidazole ring. The bending of the C–H (SP^3^) at 1457 cm^−1^ corresponds to CH_3_ substituent at the end of the imidazole ring. The C–N bending on the aromatic imidazole ring presents at 1151 cm^−1^. The peaks in the region of 600–900 cm^−1^ result from the stretching of alkyl halogen bonding or the presence of halide ions. These results are consistent with the assignment of the molecular structure of PIL-(Br) and PIL-M-(Br) [15]. Also note that there is a peak at 3141 cm^−1^ corresponding to the bending of the C–H bond on the imidazole ring. The peaks at 2981 and 2854 cm^−1^ represent the stretching of symmetric and asymmetric C–H (SP^3^) at the end substituent on the imidazole ring. The stretching at 1654 cm^−1^ corresponds to the C=N bond on the imidazole ring. The bending of the NH bond on the imidazole ring presents at 1552 cm^−1^. The bending of the C–H (SP^3^) at 1460 cm^−1^ corresponds to the CH_3_ at the end substituent on the imidazole ring. The peaks at 1345 and 1126 cm^-1^ represent symmetric and asymmetric SO_2_ bond stretching on the TFSI anion. The C–N stretching on the aromatic imidazole ring presents at 1187 cm^−1^ on the imidazole ring. The bending of symmetric CF_3_ bonding presents at 1046 cm^−1^ in the TFSI anion; the peak of 849 cm^−1^ corresponds to the stretching of an asymmetric S–N–S bond in the TFSI anion. 

### 3.3. Thermal Gravity Analysis of PILs Derived from Photopolymerization

**Figure 3:**
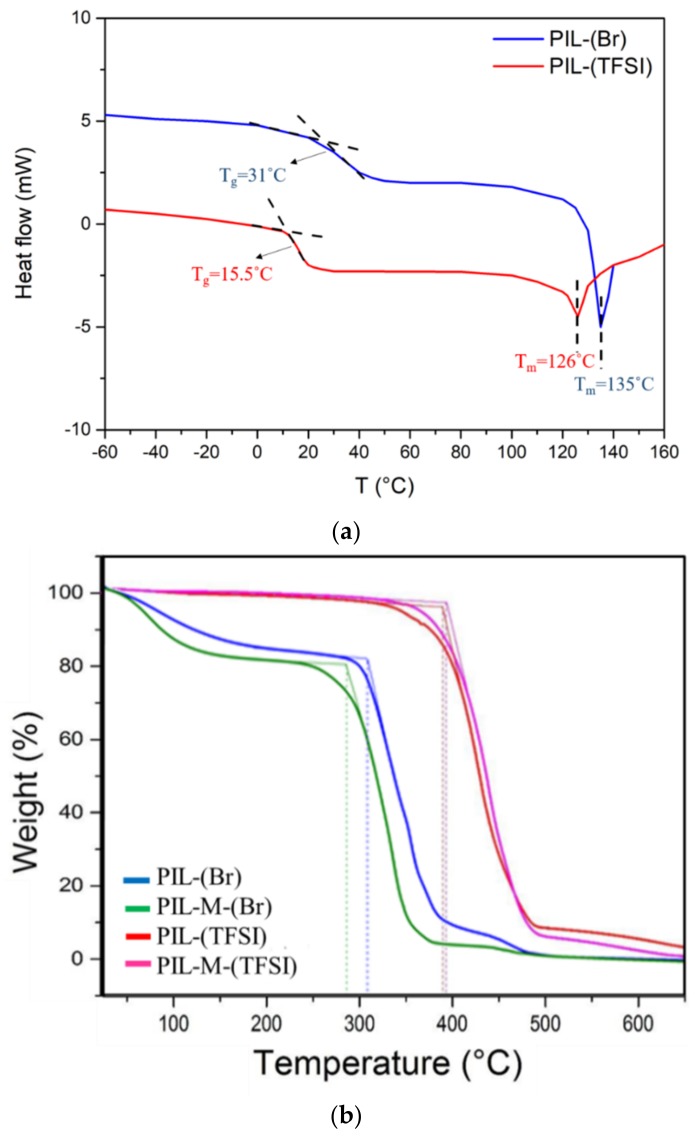
(**a**) Differential scanning calorimetric analysis of PIL-(TFSI) and PIL-(Br); (**b**) Thermal gravity analysis of PIL-(Br), PIL-M-(Br), PIL-(TFSI), and PIL-M-(TFSI).

Figure 3a shows differential scanning calorimetric analysis of PIL-(TFSI) and PIL-(Br), revealing that T_g_ (15.5 °C) of PIL-(TFSI) is lower than that (31 °C) of PIL-(Br). Also note that T_m_ (126 °C) of PIL-(TFSI) is lower than that (135 °C) of PIL-(Br). These features suggest that the molecular packing in the solid state is more efficient in presence of a Br^−^ anion than in the case of TFSI^−^. These two anions are quite different in terms of steric hindrance, symmetry and charge density, properties typically involved in solid-state lattice enthalpy and entropy. Figure 3b shows the thermal gravity analysis (TGA) of PIL-(Br), PIL-M-(Br), PIL-(TFSI), and PIL-M-(TFSI). The weight loss of the PIL-(Br) and PIL-M-(Br) samples was about 17% at 200 °C due to the evaporation of water, whereas the major weight losses occurred at 305 and 289 °C, respectively, mainly due to the thermal decomposition of PIL-(Br) and PIL-M-(Br). It can be seen that the presence of soaked MBIB resulted in a lower thermal stability of the material. Note that the major thermal decomposition temperature of PIL-(TFSI) and PIL-M-(TFSI) occurred at 387 and 391 °C, respectively. The former remained a residue of about 9.4% at about 487 °C and the latter had about 7.2% residue at about 488.5 °C. An examination of these results reveals that the two samples have very close thermal decomposition data with anion exchange of TFSI^-^ instead of Br^−^. The thermal stability of the proposed polyelectrolytes is higher in the case of the TFSI anion than the Br anion, and this is in accordance with the DSC suggestions on the energy associated to their solid state arrangements. 

### 3.4. Ionic Conductivity

**Figure 4:**
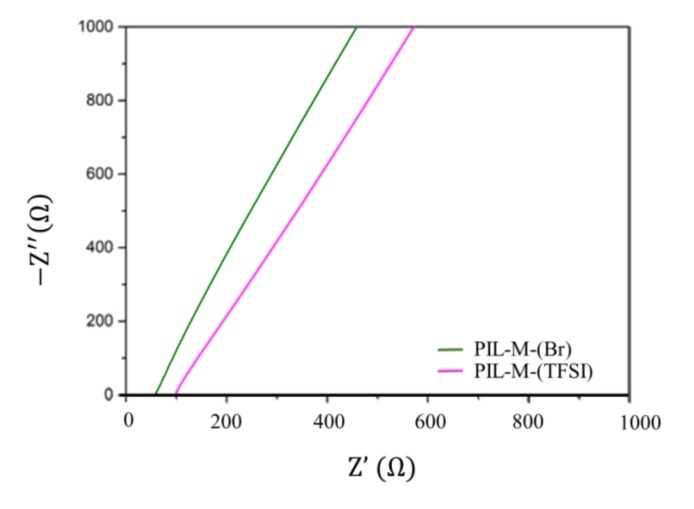
Alternating current (AC) impedance of PIL-IL blends.

Electrochemical impedance measurement can be used to calculate the ionic conductivity of the material. The ionic conductivity can be evaluated according to the following equation:

(1) $$\sigma =\frac{l}{R_{b}\times A}$$

where σ is the conductivity in S·cm^−1^, *l* is the thickness of the sample in cm, *R_b_* is the resistance of the material in Ω, and *A* is the area of the sample in cm^2^. Figure 4 shows the electrochemical impedance of PIL-M-(Br) and PIL-M-(TFSI). The value of *R_b_* was determined by the intersection point on the *x*-axis of alternating current (AC) impedance spectroscopy. Based on Equation (1), the values of ionic conductivity were 5.52 × 10^−4^ and 3.2 × 10^−4^ S·cm^−1^ for PIL-M-(Br) and PIL-M-(TFSI), respectively, at 25 °C. 

### 3.5. Cyclic Voltammogram 

**Figure 5:**
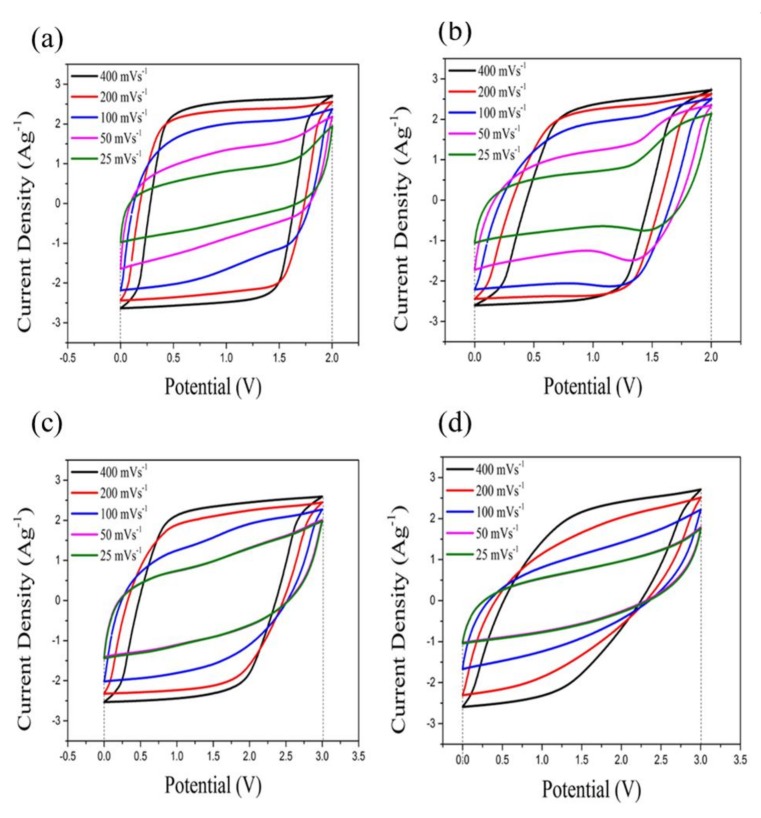
Cyclic voltammograms (CVs) of (**a**) MBIB; (**b**) PIL-M-(Br); (**c**) MBIT; and (**d**) PIL-M-(TFSI)-based supercapacitors at different scan rates.

**Figure 6:**
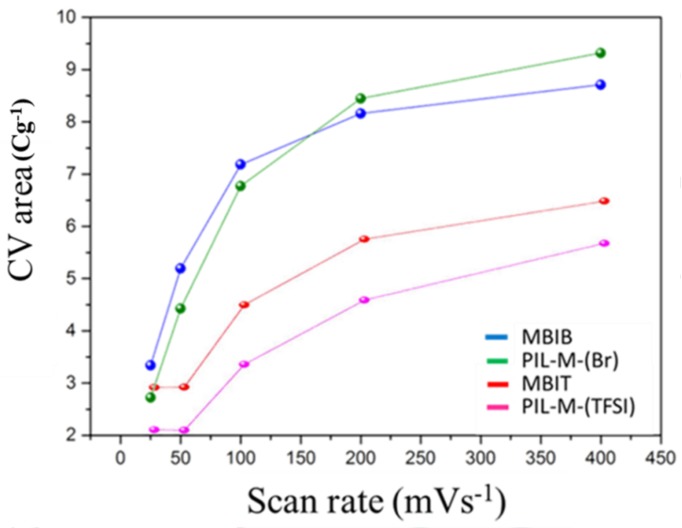
Plot of CV enclosed area against scan rate for ILs and PIL-IL blends.

Figure 5 shows cyclic voltammograms (CVs) of MBIB, PIL-M-(Br), MBIT, and PIL-M-(TFSI)-based supercapacitors. It is obvious that the shape of these CV curves is close to the rectangle, which is responsible for the characteristics of ideal electrical double layer capacitance behavior. Also note that the CV area decreases with the decreasing scan rate without a noticeable change in shape, indicating that the ions in the electrolyte can be rapidly migrated between the electrode interface and electrolyte [16]. The specific capacitance (*C_s_*) of the electrodes can be calculated from the CV curves according to the following equation:

(2) $$C_{s}=\frac{\int IdV}{Vm\Delta V}$$

where *m* is the mass of the electroactive material (g), *v* is the scan rate (V·s^−1^), ∆*V* is the potential window (V), and the integrated area under the CV curve I is the response current (A). According to Equation (1), the integrated area of the CV curve is proportional to the specific capacitance. Figure 6 shows the area enclosed by the CV curves of MBIB and PIL-M-(Br) using different scan rates. There is a small difference in specific capacitance in both electrolytes of MBIB and PIL-M-(Br) at different scan rates, implying that the capacitance values in both electrolytes of MBIB and PIL-M-(Br) are very close. This suggests that the solid-state PIL-M-(Br) electrolyte is suitable for use in a supercapacitor. However, the CV curve area of MBIB is higher than that of PIL-M-(Br) at high speed scan, indicating that the ion transport of MBIB is faster than PIL-M-(Br) because the ion mobility of liquid electrolyte is higher than that of solid electrolytes [16]. The CVs of MBIT and PIL-M-(TFSI) were similar to that of MBIB (Br) and PIL-M-(Br). In Figure 6, the CV area of MBIT is greater than that of PIL-M-(TFSI) at same scan rate, indicating that the ion transport in MBIT is significantly higher than that of PIL-M-(TFSI) because the ion mobility of the liquid electrolyte is higher than that of the solid electrolyte [16]. Especially, a larger anion size, such as TFSI^−^, has more significant effect on the ion mobility. As compared to the CVs of PIL-M-(Br) and PIL-M-(TFSI) at a scan rate of 100 mV·s^−1^, the latter has a larger potential window (3 V) than the former (2 V). However, the integrated area of the former is larger than that of the latter. This is attributed to the difference in ion conductivity between the two electrolytes, resulting from better ion transport in PIL-M-(Br) than in PIL-M-(TFSI).

### 3.6. Electrochemical Impedance Spectroscopy (EIS)

**Figure 7:**
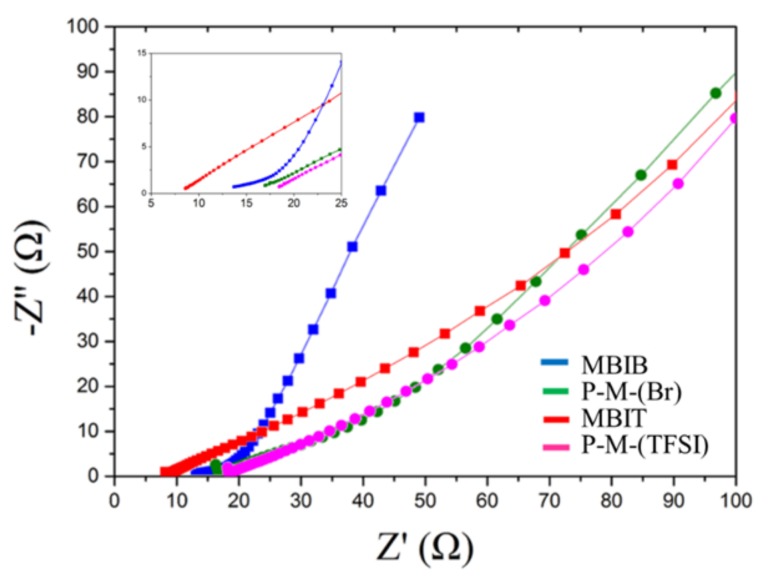
Nyquist plots of IL and PIL-IL blend-based supercapacitors.

**Figure 8:**
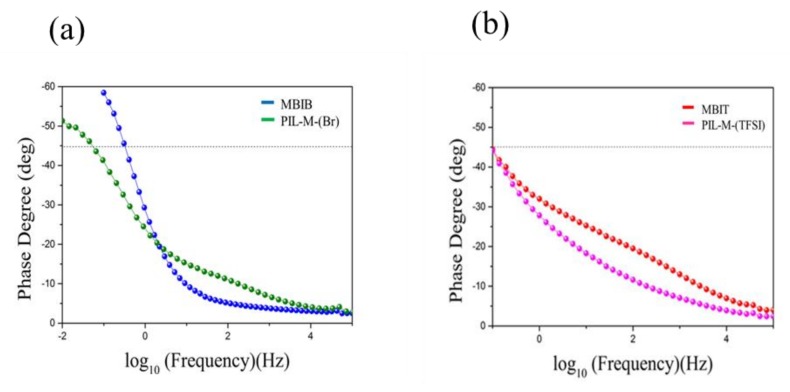
Bode plot of (**a**) MBIB and PIL-M-(Br) and (**b**) MBIT and PIL-M-(TFSI)-based supercapacitors.

Nyquist plots of MBIB, PIL-M-(Br), MBIT, and PIL-M-(TFSI)-based supercapacitors are shown in Figure 7. The equivalent series resistance (ESR) and charge transport resistance (R_ct_) are estimated in the high frequency region. The intersection of the high-frequency region with the real *x*-axis of the Nyquist plot was used to estimate the equivalent series resistance (ESR) of the electrode. The ESR values of MBIB and PIL-M-(Br) are 13 and 16.2 Ω, respectively, due to the fact that the ionic conductivity of the liquid electrolyte (MBIB) is higher than that of the solid electrolyte (PIL-M-(Br)). The ESR values of MBIT and PIL-M-(TFSI^−^) are 8.1 and 19.0 Ω, respectively. The charge transfer resistance (R_ct_) at the contact interface between the electrode and electrolyte solution forms the charge transfer limiting process corresponding to the high-frequency arc [1]. Both values of R_ct_ are very small in MBIB- and PIL-M-(Br)-based supercapacitors, corresponding to the charge transfer impedance between the interface of the electrode and electrolyte, because there are almost no electrochemical reactions involved in the charge transfer in the electric double layer capacitors, resulting in very small R_ct_. Also, both values of R_ct_ are very small in the MBIT- and PIL-M-(TFSI)-based supercapacitors. On the other hand, the straight line in the low-frequency range corresponds to the Warburg impedance, which is caused by the frequency dependence of ion diffusion and transport from the electrolyte to the electrode surfaces [1]. The MBIB and PIL-M-(Br) electrolytes exhibit the characteristics of ideal electric double layer capacitance, reflecting rapid ion diffusion in the electrolyte and adsorption onto the electrode surface. As compared to PIL-M-(Br), MBIB has a large slope in the straight line at low frequency, implying that the ions diffused in MBIB have less resistance than in PIL-M-(Br), mainly because the mass transfer in the liquid phase is easier than in the solid phase. Figure 8 shows the Bode plots of the MBIB and PIL-M-(Br)-based supercapacitors. At a phase angle of 45°, the response frequencies of MBIB and PIL-M-(Br) are 0.3 and 0.0552 Hz, whereas the *τ*_0_ values are 3.33 and 18.115 s, respectively, indicating that the MBIB has a higher power density than PIL-M-(Br). In addition, the former has a faster charge–discharge speed because the ionic conductivity of the liquid electrolyte is higher than that of the solid electrolyte, resulting in the charge transferring more quickly between electrolyte and electrode. In MBIT and PIL-M-(TFSI) systems, the response frequencies at a phase angle of 45° are the same value of 0.098 Hz and the *τ*_0_ value is the same value of 10.2 s, indicating that MBIT and PIL-M-(TFSI) have the same charge–discharge capacity under ideal capacitance conditions. Also note in Figure 7, that the slope of PIL-M-(Br) is larger than that of PIL-M-(TFSI) in the low frequency range, implying that the former has less resistant ion diffusion than the latter. This is attributed to the fact that the ionic conductivity of PIL-M-(Br) is greater than that of PIL-M-(TFSI). Table 3 summarizes the data obtained by the EIS analysis of PIL-M-(Br) and PIL-M-(TFSI)-based capacitors. An examination of Table 3 reveals that the ESR values of PIL-M-(Br) are lower than those of PIL-M-(TFSI). This reflects the fact that the former has lower solution resistance than the latter. In addition, the *τ*_0_ values of PIL-M-(Br) are higher than those of PIL-M-(TFSI), suggesting that the latter capacitor has a higher charge–discharge rate than the former in the ideal state.

### 3.7. Galvanic Charge–Discharge (GCD)

**Figure 9:**
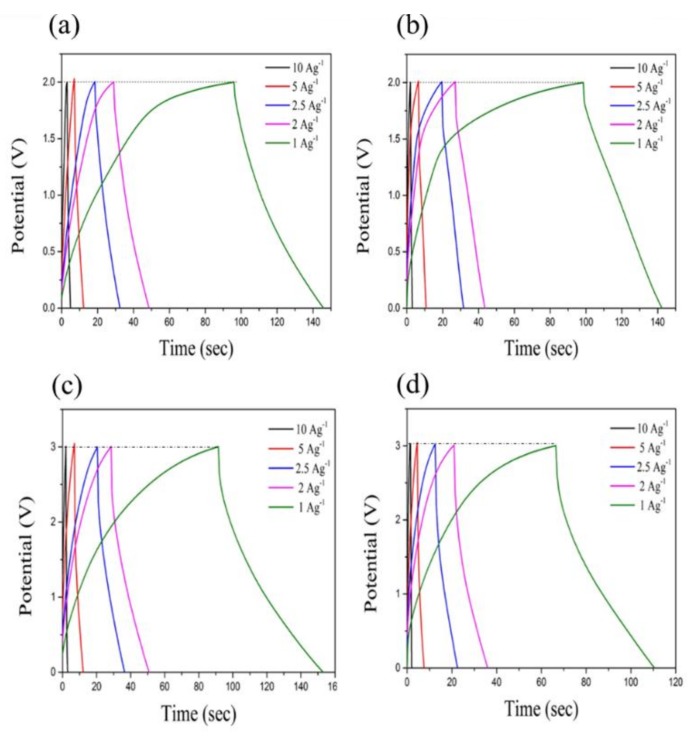
Galvanic charge–discharge curves of (**a**) MBIB; (**b**) PIL-M-(Br); (**c**) MBIT; and (**d**) PIL-M-(Br)-based supercapacitors at different current densities.

**Figure 10:**
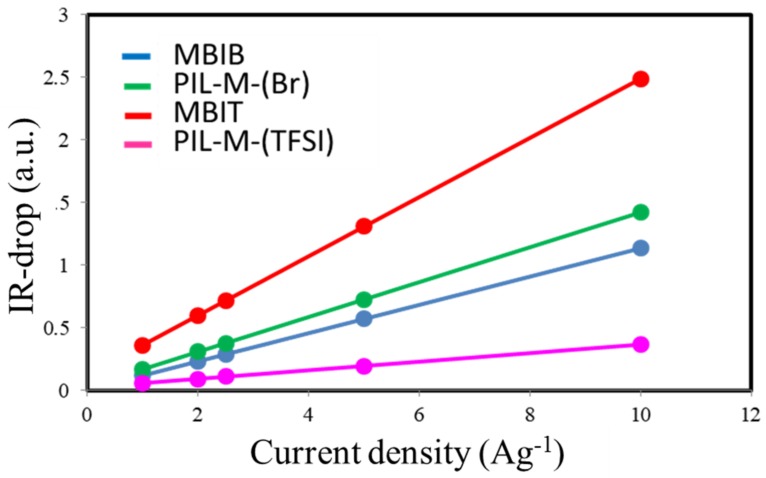
Plot of IR-drop against current density for ILs and PIL-IL blends.

**Figure 11:**
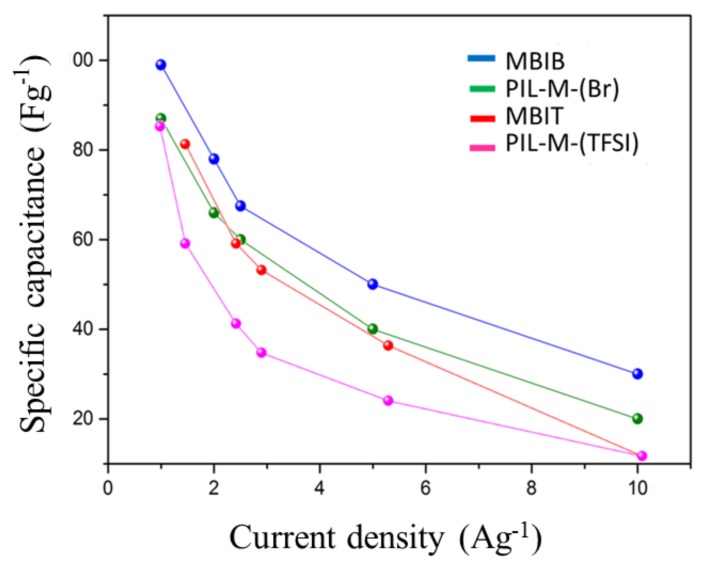
Plot of specific capacitance against current density for ILs and PIL-IL blends.

Figure 9 shows the galvanostatic charge–discharge curves at current densities of 10, 5, 2.5, 2 and 1 A·g^−1^ in the potential window of 0–2 V for MBIB- and PIL-M-(Br)-based supercapacitors. These charge–discharge curves have a symmetrical triangular shape, indicating that the ideal characteristics of the electrical double-layer capacitor (EDLC) provide these electrodes with excellent electrochemical reversibility [2]. However, different charge and discharge current densities did not change the line shape, demonstrating fast charge–discharge kinetic behavior [17]. Figure 10 shows the voltage (IR drop) at different charge–discharge current densities, revealing that the voltage increases with an increasing charge–discharge current density. This arises from the fact that the charge diffusion rate cannot match the rapid increase in current densities [18,19]. In addition, the slope of the straight line represents the bulk resistance. The bulk resistance of the MBIB-based capacitor is less than that of the PIL-M-(Br)-based capacitor, because the ionic conductivity of MBIB is greater than that of PIL-M-(Br). The specific capacitance values are calculated from the results of the constant current charge–discharge. Figure 11 shows the specific capacitance values of MBIB- and PIL-M-(Br)-based capacitors at different current densities. The specific capacitance of both systems decreases with an increasing charge–discharge current density. This is because the increase of the current density causes the increase of ion-diffusion resistance in the electrode material, leading to the decrease of the ion diffusion rate in the electrolyte, thereby lowering the capacitance. The specific capacitance of MBIB is slightly larger by about 10% than that of PIL-M-(Br). One explanation for this result is that MBIB has a higher ionic conductivity than PIL-M-(Br), resulting in an MBIB solution resistance smaller than PIL-M-(Br). Moreover, the mass transfer rate of MBIB is faster than that of PIL-M-(Br), which makes MBIB ions easier to adsorb and diffuse on the electrode material. The potential window (3 V) of PIL-M-(TFSI) is larger than that (2 V) of PIL-M-(Br). In addition, the specific capacitance was plotted against the current density as shown in Figure 11, revealing that the specific capacitance of the PIL-M-(Br)-based capacitor is higher than that of the PIL-M-(TFSI)-based capacitor. These results can be supported by a lower IR drop of PIL-M-(Br^−^ system as compared PIL-M-(TFSI) (Figure 11), suggesting that the ionic conductivity of PIL-M-(Br) is greater than that of PIL-M-(TFSI).

**Figure 12:**
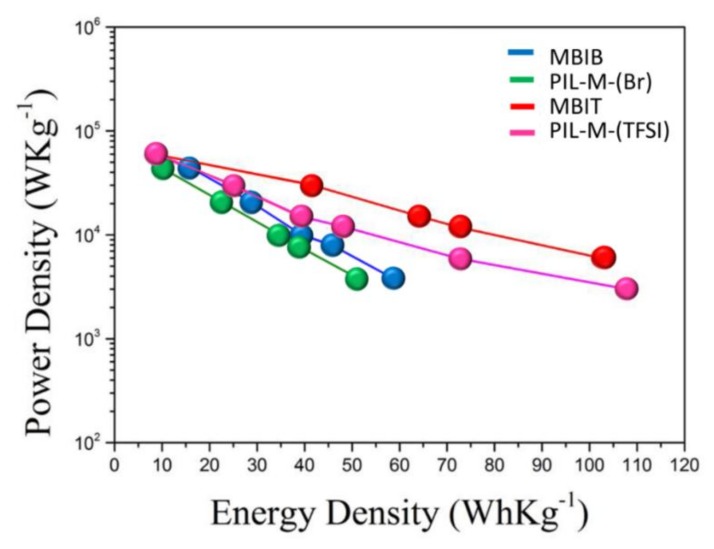
Ragone plot of MBIB and PIL-M-(Br^−^)-based supercapacitors.

In Figure 12, specific energy density (Wh·kg^−1^) and specific power density (W·kg^−1^) can be calculated to obtain the Ragone plot. The MBIB-based supercapacitor has a slightly higher specific power density at the same specific energy density, and vice versa, indicating that the MBIB-capacitor can store a higher amount of electricity in a unit mass, whereas it can also store or release a higher power in a unit of time, but there are no large differences between the two systems of MBIB- and PIL-M-(Br)-based supercapacitors. Also note that the PIL-M-(Br)-based supercapacitor has a higher specific power density in relation to the PIL-M-(TFSI)^-^-based supercapacitor at the same specific energy density. In contrast, the PIL-M-(TFSI)-based supercapacitor has a higher specific energy density at the same specific power density. 

**Figure 13:**
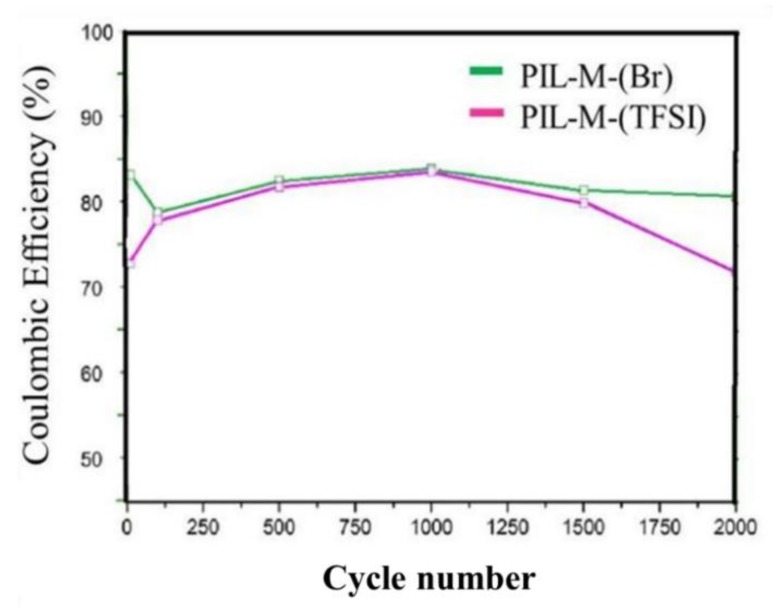
Cycle life of PIL-Il blend-based supercapacitors.

Figure 13 shows cycle life of the Coulombic efficiency in PIL-M-(Br)- and PIL-M-(TFSI)-based supercapacitors, revealing that the Coulomb efficiency maintains at 80% and 72%, respectively, after 2000 cycles. This is attributed to the fact that ions accumulate in the pores of the electrode material, reducing the redox process of the electrolyte. After repeated cycles, the electrolyte may deteriorate to decrease the ionic conductivity of the electrolyte with the increase in electrolyte resistance [17,20,21,22,23]. The PIL-M-(TFSI)-based supercapacitor has a similar tendency.

## 4. Conclusions

The ionic liquid polymer blends PIL-M-(Br) and PIL-M-(TFSI) had thermal stability with higher decomposition temperatures of 305 and 391 °C, respectively. The PIL-M-(Br) and PIL-M-(TFSI) had high ionic conductivity of 0.55 and 0.32 mS·cm^−1^, respectively, which were very suitable for use in carbon-based symmetric supercapacitors, with the potential windows of 2 V and 3 V with respect to PIL-M-(Br) and PIL-M-(TFSI), respectively. Carbon-based symmetric supercapacitors involving the respective electrolytes PIL-M-(Br) and PIL-M-(TFSI) had a high power density and energy density of 59.9 and 40 kW·kg^−1^, and 48 and 107 Wh·kg^−1^, respectively. The PIL-M-(Br)-based supercapacitor had a higher specific capacitance than PIL-M-(TFSI) with specific capacitances of 87 and 58 F·g^−1^, respectively, at a charge–discharge current density of 1 A·g^−1^. In contrast, the values of the MBIB and MBIT ionic liquids were 99 and 82 F·g^−1^, respectively. After 2000 cycles of charge–discharge testing, the efficiency decay of solid state PIL-M-(Br) and PIL-M-(TFSI)-based supercapacitors was 12 and 29%, respectively. The time constant (τ_o_) of PIL-M-(Br) is longer than that of PIL-M-(TFSI), suggesting that the latter has a better charge–discharge rate under ideal capacitors.

## Figures and Tables

**Table 1:** Preparation process of the ionic liquid monomer.

Component	Reaction Scheme
MBIB	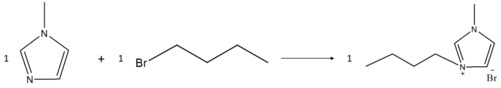
BVIB	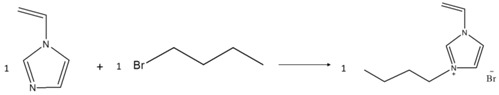
BDVIB	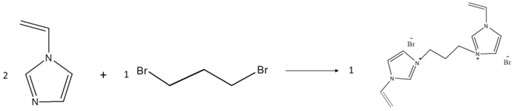
MBIT	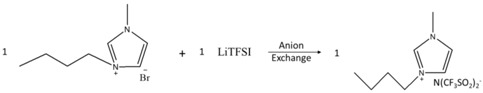
BVIT	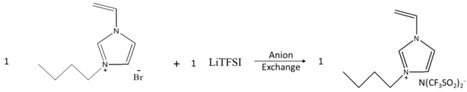
BDVIT	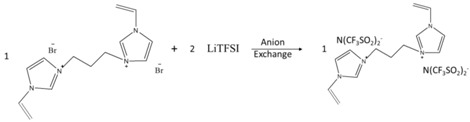
PIL-(Br)	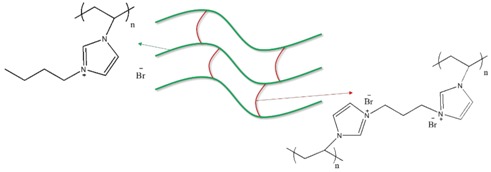
PIL-(TFSI)	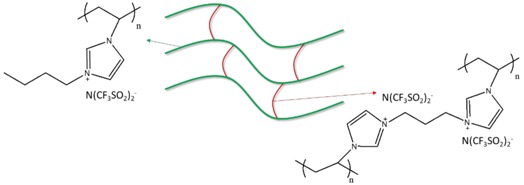

**Table 2:** IR-ATR observed wavelengths, in cm^−1^, of the absorption bands detected in the spectra of PIL-(Br), PIL-M-(Br), PIL-(TFSI) and PIL-M-(TFSI).

No.	PIL-(Br)	PIL-M-(Br)	Assignment	PIL-(TFSI)	PIL-M-(TFSI)	Assignment
1	3077		C–H bending	3141		C–H bending
2	2981 2932 2869		C–H streching	2981 2854		C–H streching
3	1650		C=C strehing	1654		C=N streching
4	1546		C=N bending	1552		C=N bending
5	1457		C–H bending	1460		C–H bending
6	1151		C–N bending	1345 1126		O=S=O streching
7	600–900		bromide	1187		C–N streching
8				1046		CF bending
9				849		S–N–S

**Table 3:** EIS data of PIL-M-(Br)- and PIL-M-(TFSI)-based supercapacitors.

Substance	ESR (Ω)	$\tau_{0}$
PIL-M-(Br)	16.2	18.1
PIL-M-(TFSI)	19.0	10.2
